# Effects of diabetic retinopathy on longitudinal morphological changes in AMD-associated type 1 macular neovascularization

**DOI:** 10.1038/s41598-023-43635-4

**Published:** 2023-09-28

**Authors:** Pasquale Viggiano, Luca Landini, Maria Oliva Grassi, Giacomo Boscia, Enrico Borrelli, Giancarlo Sborgia, Giovanni Alessio, Francesco Boscia

**Affiliations:** 1 Department of Translational Biomedicine Neuroscience, University of Bari “Aldo Moro”, Piazza Giulio Cesare, 11, Bari, Italy; 2 Ophthalmology Department, San Raffaele University Hospital, Milan, Italy

**Keywords:** Biomarkers, Medical research, Molecular medicine

## Abstract

The purpose of this study was to investigate the effect of diabetic retinopathy (DR) on longitudinal morphological changes in AMD-associated type 1 macular neovascularization using optical coherence tomography angiography (OCTA). We enrolled fifty treatment-naïve eyes with a diagnosis of exudative AMD and type 1 MNV. Twenty of 50 eyes were affected by mild DR. En face OCT angiography were examined for the MNV lesion area (mm^2^), the MNV flow area (mm^2^), the central macular thickness (CMT) and the BCVA. The OCTA acquisition was performed at the following visits: (i) before the loading phase (LP) of intravitreal injection of aflibercept (T1), and (ii) 1 month after the last intravitreal injection of loading phase comprising 3 monthly injections (T2). All morpho-functional parameters showed a significantly change at T2 compared to T1 values in both groups. Furthermore, we found a greater MNV area reduction after LP in eyes without DR (P = 0.023). With regard to the remaining parameters, no significant changes were found between two groups (P > 0.05). Our analysis revealed a less MNV area reduction after loading dose of anti-VEGF therapy in eyes affected by diabetic retinopathy.

## Introduction

Age-related macular degeneration (AMD) represents the first cause of visual impairment in aged people^1^. The pathogenesis of AMD is considered to be multifactorial. Although previous studies have evaluated several demographic, medical or genetical risk factors for AMD, the majority remains controversial^2^. Cardiovascular disease and AMD have been hypothesized to share common pathways^3^. Diabetes mellitus (DM), recognized as the dominant cause of cardiovascular disease, has also been correlated to AMD. Furthermore, the relationship between DM and AMD is controversial. Even though some authors reported diabetes as a risk factor for AMD^4^ (i.e. specially for neovascular AMD (nAMD)), other studies did not find any correlation between these two disorders^5^, while others found diabetes as a protective factor^6^. Importantly, diabetic retinopathy (DR) and nAMD share treatment options, as anti-VEGF intravitreal injections are used to treat both AMD-associated macular neovascularization (MNV) and diabetic macular oedema^7,8^.

With the recent advent of optical coherence tomography angiography (OCTA), an accurate assessment of the retinal and choroidal layers at a high resolution has been allowed in vivo^9,10^. Importantly, OCTA has provided the ability to assess anatomic characteristics of MNV and its longitudinal changes in response to anti-VEGF treatment in patients with neovascular AMD^7,11^. The latter studies have demonstrated an overall reduction in MNV size after anti-VEGF therapy^7,12,13^. On OCTA, type 1 MNV may be visualized in two different patterns: (i) the “sea fan” pattern featured by vessels branching from one side of the MNV and; (ii) the “medusa” pattern with neovessels branching outward from the core of the lesion^14^.

Nevertheless, the current literature lacks information regarding the influence of diabetes and diabetic retinopathy on AMD-associated MNV. Therefore, our OCTA study aimed at assessing whether the presence of diabetes and DR may impact on the baseline characteristics of treatment-naïve AMD-associated type 1 MNV. More importantly, we investigated the longitudinal changes occurring to type 1 MNV during anti-VEGF therapy in order to understand whether the presence of diabetes and DR may impact on such modifications.

## Results

### Characteristics of patients included in the analysis

A total of 50 eyes of 50 patients with naïve nAMD-associated type 1 macular neovascularization were included in the study. Twenty-three patients were men, and 27 patients were women. The mean age of diabetic group was 76.3 ± 5.1 years (range = 55–85 years). The mean age of no diabetic group was 77.8 ± 5.8 years (range = 56–85 years). The comparison between two groups did not display significantly changes (P ≥ 0.05). The characteristics of subjects included in the analysis are shown in Table 1. Subjects were evaluated at the following visits: (i) “T1 visit” corresponding the day before the first anti-VEGF, and (ii) “T2 visit” corresponding to the examination performed 1 month after the last injection within the loading phase. The outcome measures encompassed the entire CNV lesion area, the CNV flow area, central macular thickness, and the alteration in BCVA.

**Table 1:** The clinical characteristics of subjects included in the analysis.

Variables	Diabetic group	No diabetic group	P value
Number	20	30	0.477
Age (years)	76.3 ± 5.1	77.8 ± 5.8	0.248
Gender (female, %)	13 (65%)	14 (46%)	0.763
Cardiovascular disease* (n, %)	5 (25)	4 (13)	0.678

Data are presented as Mean ± SD.

*History of heart attack or stroke.

### BCVA and central macular thickness analysis

The BCVA significantly improved in both groups after loading dose of aflibercept. Mean ± SD BCVA of diabetic eyes was 0.49 ± 0.13 logMAR at T1 and 0.33 ± 0.17 logMAR at T2 (p = 0.003). Mean ± SD BCVA of no diabetic eyes was 0.51 ± 0.16 logMAR at T1 and 0.34 ± 0.15 logMAR at T2 (p = 0.002) (Table 2).

**Table 2:** Morpho-functional results.

	Diabetic group			No diabetic group		
	T1	T2	p value	T1	T2	p value
BCVA (logMAR)	0.49 ± 0.13	0.33 ± 0.17	0.003	0.51 ± 0.16	0.34 ± 0.15	0.002
CMT (μm)	292.4 ± 42.23	238.5 ± 33.32	0.001	288.3 ± 41.16	266.71 ± 34.94	0.001
MNV lesion area (mm^2^)	1.31 ± 1.02	1.03 ± 0.91	0.001	1.32 ± 1.04	0.89 ± 0.83	0.001
MNV flow area (mm^2^)	0.86 ± 0.66	0.59 ± 0.54	0.001	0.78 ± 0.59	0.52 ± 0.48	0.004

Data and comparisons (T1 vs T2).

Data are presented as mean ± SD. BCVA best-corrected visual acuity.

T1 before loading anti-VEGF therapy; T2 after loading anti-VEGF therapy.

CMT showed statistically significant changes in both groups between following visits. In detail, the CMT of diabetic group was 292.4 ± 42.23 μm at T1 and 238.5 ± 33.32 μm at T2 (p = 0.001). Mean ± SD CMT of no diabetic group was 288.3 ± 41.16 μm at T1 and 266.71 ± 34.94 μm at T2 (P = 0.001). Regarding BCVA and CMT analysis, the comparison between two groups did not display significantly changes (P ≥ 0.05) (Table 2).

### Macular neovascularization OCTA analysis

**Figure 1:**
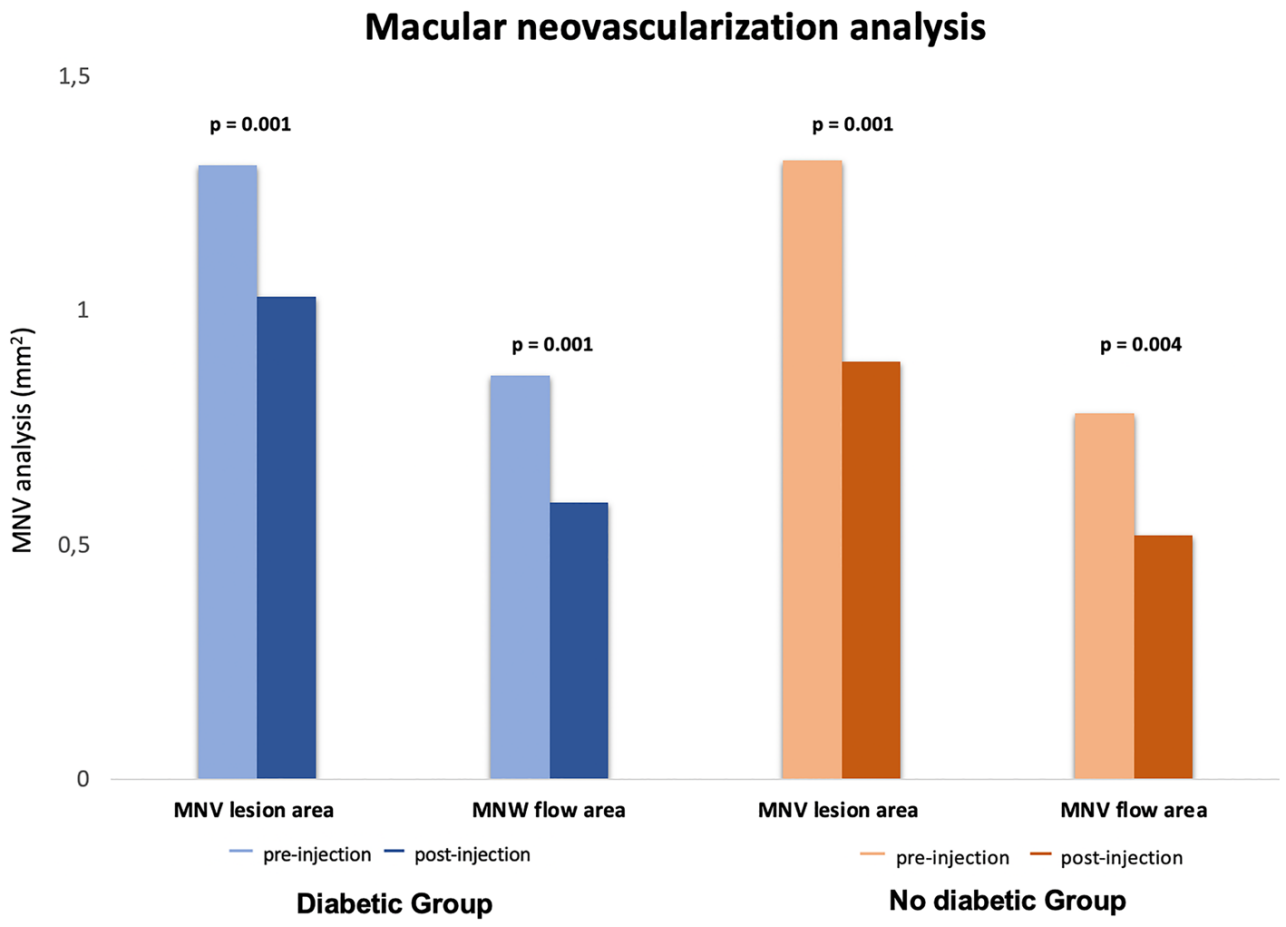
Graph showing macular neovascularization modifications in both groups after intravitreal therapy. The MNV lesion area and the MNV flow area were significantly reduced in both groups after LP of aflibercept.

The MNV lesion area and the MNV flow area were significantly reduced in both groups after LP of aflibercept (Fig. 1). In detail, the MNV lesion area of diabetic group was 1.31 ± 1.02 mm^2^ at T1 and 1.03 ± 0.91 mm^2^ at T2 (P = 0.001). Mean ± SD MNV lesion area measured of no diabetic group was 1.32 ± 1.04 mm^2^ at T1 and 0.89 ± 0.83 mm^2^ at T2 (P = 0.001) (Table 2). Importantly, the diabetic eyes displayed a lower reduction of the MNV lesion area compared with no diabetic eyes (P = 0.023) (Table 3). The intraclass correlation coefficient was 0.96 (95% confidence interval [CI] 0.931–0.962) for the MNV area quantification.

**Table 3:** Comparison between diabetic vs no diabetic eyes.

	Diabetic group (n = 20)	No diabetic group (n = 30)	P value
BCVA (logMAR)	13.5 ± 10.9	13.3 ± 9.7	0.934
CMT (μm)	− 14.1 ± 11.5	− 15.1 ± 11.9	0.769
MNV lesion area (mm^2^)	− 24.8 ± 18.7	− 35.4 ± 17.6	0.023
MNV flow area (mm^2^)	− 32.2 ± 27.7	− 32.9 ± 23.1	0.148

Data are delta percentages (Mean ± SD). BCVA best-corrected visual acuity CMT central macular thickness.

*MNV* macular neovascularization, *n* number of patients.

T1 before loading anti-VEGF therapy; T2 after loading anti-VEGF therapy; Friedman non-parametric test was performed to obtain P-values.

Likewise, the MNV flow area measured of diabetic eyes was 0.86 ± 0.66 mm^2^ at T1 and 0.59 ± 0.54 mm^2^ at T2 (P = 0.001) and MNV flow area measured of no diabetic eyes was 0.78 ± 0.59 mm^2^ at T1 and 0.52 ± 0.48 mm^2^ at T2 (P = 0.004) (Table 2). The comparison between two groups did not display significantly changes (P ≥ 0.05) (Table 3).

## Discussion

In this prospective OCT and OCTA study, we examined the effect of DR on longitudinal morphological and functional changes in AMD-associated MNV type 1 after the loading phase of anti-VEGF treatment. We found that mild NPDR eyes responded differently to nAMD treatment. In particular, the diabetic group showed limited contraction of the MNV lesion area after intravitreal therapy, which should be taken into consideration because OCTA-based metrics are currently used for the management of nAMD and DR.

Diabetes-related alterations in the structure and function of the RPE and the choroidal circulation have been hypothesized to increase the nAMD risk^15^. Previous histologic papers have showed thickening of the CC walls, luminal narrowing, dropout of the CC and thickening of the Bruch membrane in long-lasting diabetic eyes^15,16^. These factors may predispose to the development of nAMD. However, there is poor evidence of the DR impact on treatment response in eyes affected by nAMD since data from previous studies are inconsistent.

Recently, several authors have investigated morpho-functional changes after different VEGF treatments^17,18^. CMT is commonly used for objective documentation of progress, whereas the BCVA represents the main outcome parameter of numerous studies. We observed in both groups a significant BCVA improvement after LP treatment confirming the findings of previous studies^19,20^. Similarly, our different populations included in this study showed significant CMT reduction after intravitreal injections of aflibercept. Although the influence of central macular thickness on the baseline BCVA is known^21^, its importance during the therapeutic regimen is debatable. Furthermore, we did not observe BCVA and CMT difference between two groups, suggesting no functional DR impact on the eyes affected by nAMD.

Nevertheless, at the best of our knowledge, no study focused on the MNV modifications after VEGF antagonist treatment in eyes affected by nAMD and mild NPDR. OCTA allows the clinician to capture depth-resolved information and definite images of MNV in nAMD^22,23^. For this reason, by means of OCTA, we analyzed the MNV size and flow area in both groups. We showed a reduction of the MNV area after LP aflibercept injections, and this reduction was visible in both groups. In accord with our results, Coscas et al.^24^ and Muakkassa et al.^25^ confirmed a reduction of the lesion area of type I and type II neovascularization after different anti-VEGF drugs, suggesting the importance of MNV size as a useful OCTA parameter in the analysis of response or lack of response to treatment. Interestingly, diabetic eyes was characterized by a minor contraction in MNV area compared with no diabetic eyes. We might hypothesize two co-existing mechanisms behind the latter finding: (1) the presence of DR in eyes affected by nAMD could lead to higher levels of VEGF and therefore a worse response to treatment in terms of MNV downsizing; (2) DR is known to be associated with choroidal hypoperfusion that might contribute to the MNV persisting as a mechanism of defense to the RPE and outer retina ischemia.

Choi et al.^26^ reported that diabetes mellitus is a risk factor for early AMD compared with no early AMD. In their meta-analysis, Chen and colleagues^27^ suggested that diabetes represents a risk factor for AMD, stronger for late AMD than earlier stages. On the contrary, in a South Indian Population study^28^, the authors observed that DR is a protective factor for AMD in subjects with DM. The conflicting findings might be due to different ophthalmic and systemic variables examined. Therefore, we investigated the DR risk in response to nAMD treatment using OCTA technology.

In addition, we also evaluated the MNV flow area changes in both groups showing a reduction in MNV flow area after LP anti-VEGF treatment. Our data confirmed previous results, indeed Mastropasqua et al.^23^ displayed the MNV flow area decrease in a prospective study enrolling 15 naive patients with nAMD. Of note, we did not find MNV flow difference between two groups. Our results give credit to the theory that the treatment leads to a reduction in the number and the perfusion of the smallest pathological vessels while the larger trunks remain well-perfused after the anti-VEGF treatment^24^, even in nAMD complicated by DR.

Significantly, a notable feature of neovascular AMD eyes is the often observed disparity between anatomical structures and visual capabilities. This discrepancy is frequently attributed to changes in the inner and outer retina as well as the retinal pigment epithelium (RPE), which can persist even after successful fluid resolution^29^.

Our study has limitations to consider when interpreting our findings. Our sample size was relatively small. Moreover, our study lacks a control group, implying that differences during the follow-up could have been occurred even if the anti-angiogenic therapy was not applied. Another major limitation is that we used spectral domain OCTA which utilizes shorter wavelength light in comparison with swept source OCT angiography, resulting in less signal pass-through the RPE. Additionally, our analysis has not been adjusted for potential confounding factors related to systemic and ocular parameters. Finally, we followed up the patients up to 1 month after the LP of aflibercept. This limit did not allow us to evaluate long-term MNV changes.

However, also the strengths of our study should be kept in mind. In particular, we enrolled only patients with type 1 MNV naïve, as this type of MNV may be easily investigated with OCTA, even in terms of quantitative assessment. Furthermore, at the best of our knowledge, no study analyzes nAMD eyes affected even by mild NPDR and evaluates morpho-vascular and functional changes after anti-VEGF treatment. We showed, by means of OCTA, a different response to intravitreal therapy in terms of MNV area lesion in DR group, indicating DM as risk factor to consider in patients affected by nAMD during anti-angiogenic therapy.

In conclusion we provide the first fully integrated study of the effects of DR on longitudinal morphological and functional changes in AMD-associated type 1 MNV undergoing 3 monthly aflibercept intravitreal injections. Using OCTA, we showed a different response to intravitreal therapy in terms of MNV area lesion in DR group. In particular, diabetic eyes were characterized by a minor contraction in MNV area compared with no diabetic eyes, the latter feature indicating DM as risk factor to consider in patients affected by nAMD during anti- angiogenic treatment. These results provide evidence that the DR impact might play a fundamental role not only in the development and progression of MNV but also in evaluating reaction to anti-angiogenic therapy. Future larger studies using SS-OCTA and longer follow-up are needed to support our preliminary findings.

## Methods

### Study participants

This prospective cohort study analyzed fifty eyes with a diagnosis of exudative neovascular AMD and type 1 MNV naïve undergoing a loading dose of anti-VEGF treatment (3 monthly anti-VEGF injections) at the retina service between September 2021 and May 2022. This study observed the tenets of the Declaration of Helsinki and was approved by the institutional review board of Department of Translational Biomedicine Neuroscience, University of Bari “Aldo Moro”. The patients gave their written informed consent to participate in the study.

Enrolled patients were divided according to the presence of diabetes and diabetic retinopathy yielding to a group of 30 individuals without evidence or history of diabetes, and 20 out of 50 subjects with diabetes and mild NPDR according to the modified ETDRS retinopathy severity scale^30^. Mild NPDR was defined as the presence of at least one microaneurysm and/or mild hemorrhages. We divided the subjects in two groups: “diabetic group” corresponding to eyes affected by nAMD and DR (20 eyes); no diabetic group corresponding to eyes affected only by nAMD (30 eyes).

Exclusion criteria were: (i) presence of type 2 or type 3 MNV, (ii); presence of significant cataract (iii) infection or inflammation of both eyes; (iv) any optic neuropathy, including glaucoma, (v) presence of other comorbid macular diseases; (vi) history of anti-VEGF injection or retinal laser therapy in the study eye; (vii) history of myocardial infarction or cerebrovascular disease within the last 6 months; (viii) myopia greater than 3.00 diopters and (ix) positive history for any retinal treatment^31^; and (x) neurodegenerative diseases (e.g., Alzheimer disease or Parkinson disease). Moreover, poor quality images with either significant incorrect segmentation and/or motion artifact were not included in the analysis^32^. Images with inadequate signal strength (< 6/10) were excluded.

### Study protocol

At baseline, all patients underwent a complete ophthalmologic examination, including BCVA, intraocular pressure evaluation, and dilated ophthalmoscopy. Furthermore, all patients were tested by means of XR Avanti AngioVue OCTA (Optovue Inc, Fremont, CA). Subjects were evaluated at the following visits: (i) “T1 visit” corresponding the day before the first anti-VEGF, and (ii) “T2 visit” corresponding to the examination performed 1 month after the last injection within the loading phase.

All patients were treated with aflibercept IVI within 3 days from the baseline assessment (T1). Then, aflibercept was administered monthly for a total of 3 injections. At each follow-up visit, patients received both a complete ophthalmologic evaluation, to assess changes in BCVA, and OCT and OCTA imaging to evaluate quantitative changes.

Outcome measures included whole CNV lesion area, the CNV flow area, central macular thickness and the change in BCVA.

### Imaging acquisition and processing

#### Vascular layer segmentation

Structural OCT and OCTA imaging was performed using the RTvue- XR Avanti (Optovue) device that uses an A-scan rate of 70,000 scans per second. The Optovue Avanti system obtains 2 consecutive OCTA volume scans (vertical and horizontal), and then combined to diminish motion artifacts^32^. A 3 × 3 mm OCTA scan was captured for each eye using FastTrac motion correction software to eliminate segmentation and/or shadowing artefacts.

#### Macular neovascularization analysis

**Figure 2:**
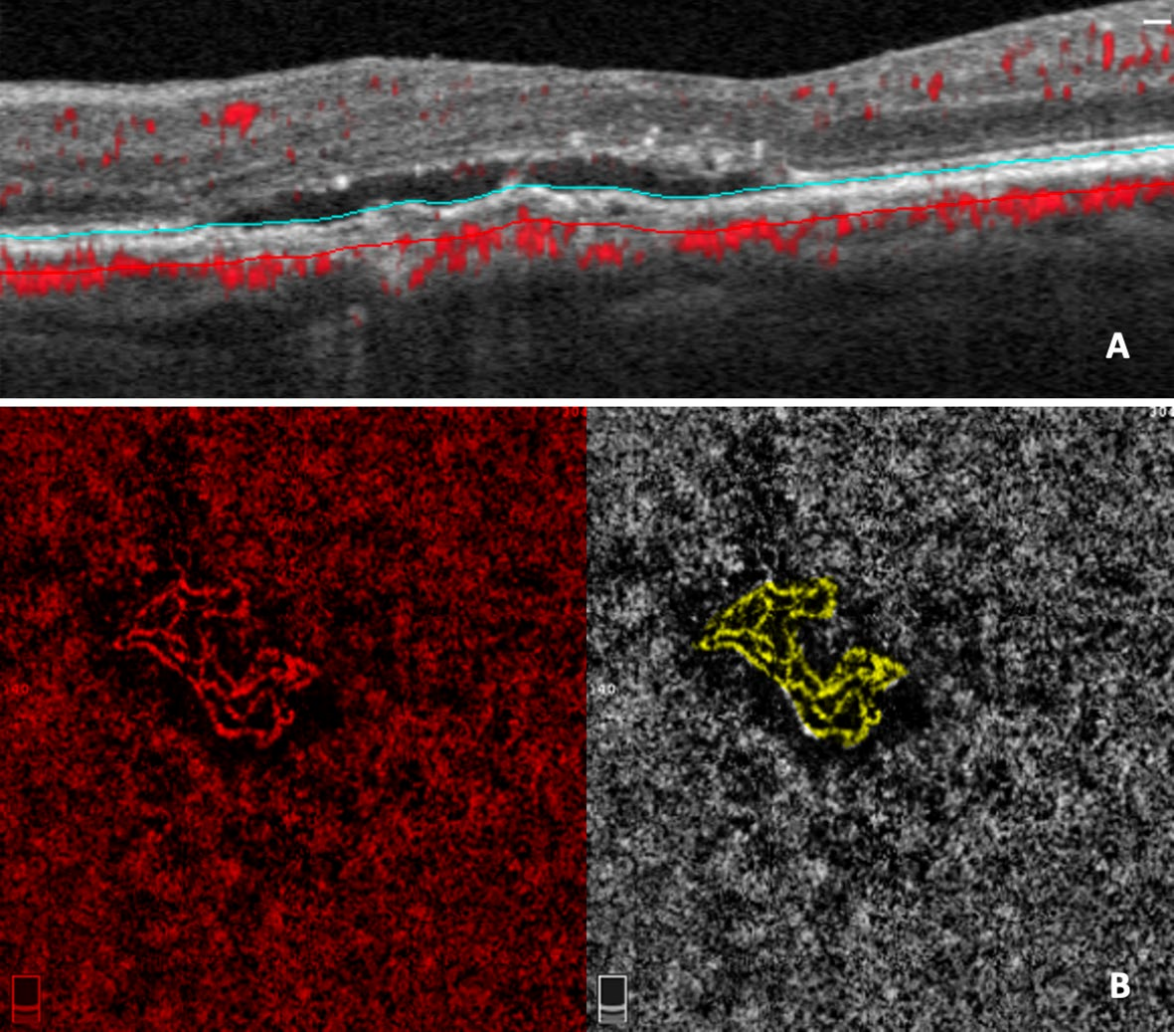
(**A**) SD-OCT image of patient affected by AMD-associated MNV type 1 at baseline evaluation. To evaluate the type 1 MNV, a retinal layer, with the inner border at the level of the outer aspect of the inner nuclear layer and the outer border at the level of Bruch membrane, was set. Then, the inner and outer border levels were manually changed finely to include all the area suspicious for MNV. (**B**) OCTA en face image of the neovascular complex. Color-coded flow analysis with manually drawn lesion borders. The MNV borders define the ROI of which the software automatically outputted the MNV lesion area (the entire area inside the drawn ROI) and the MNV flow area (the area of flow in the user-defined MNV lesion area) values.

**Figure 3:**
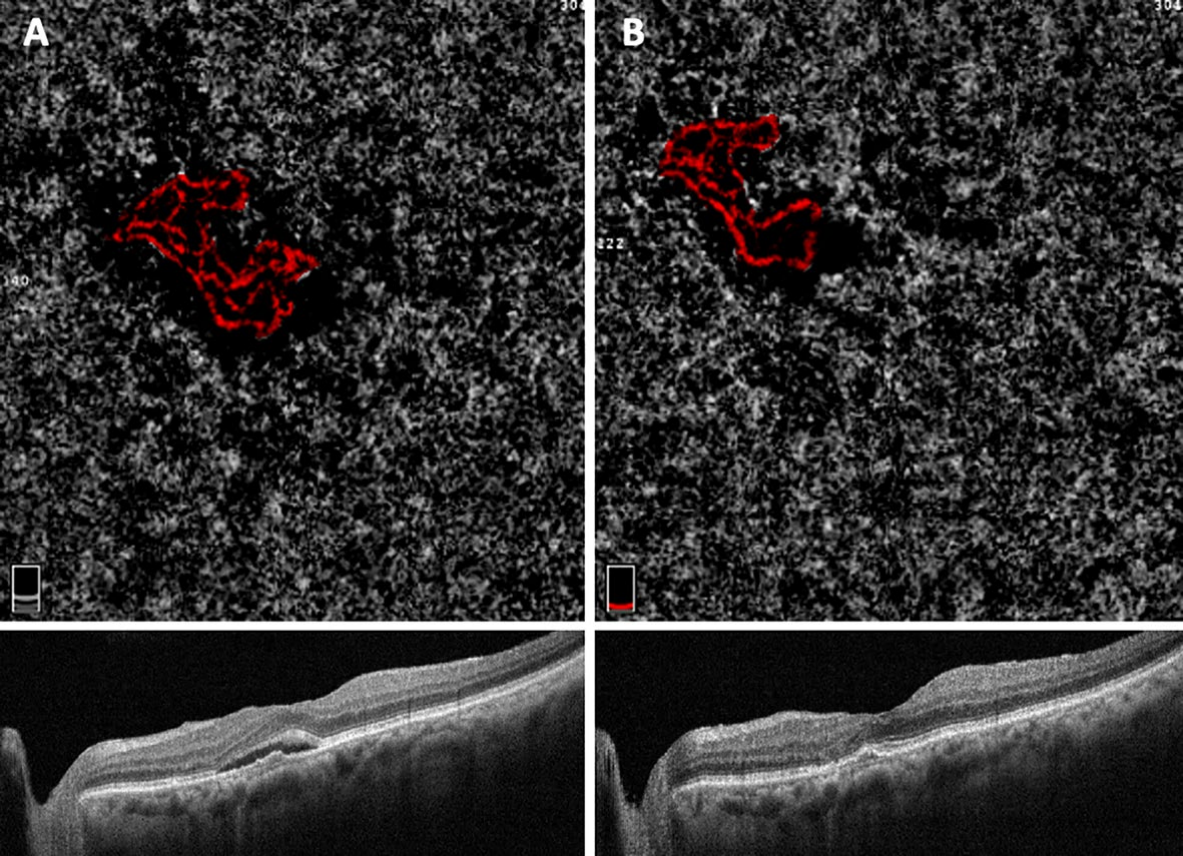
OCT and OCTA scans, from an enrolled patient affected by Type 1 MNV in AMD and treated with 3 monthly aflibercept IVI. Scans were acquired at baseline visit and 1 month after loading-dose after aflibercept injections. The figure shows the MNV changes throughout the follow-up. Left panel (**A**): before therapy; Right panel (**B**): after therapy.

To visualize the whole MNV, the methodology displayed by de Carlo et al.^22^ was used. Briefly, manual segmentation was used to capture the choriocapillaris en face (CC-OCTA) image (slab 30 μm thick beginning 31 μm posterior to the RPE—Bruch’s membrane complex). Therefore, the outer and inner boundary levels have been finely modified to include the entire MNV area, as visualized on the corresponding OCT B-scan images (Fig. 2A). CC areas beneath superficial retinal vasculature were eliminated from the analysis to avoid potential projection or shadows artefacts***.*** Finally, on the CC en face image, we drew an ROI following the MNV perimeter. The borders of the MNV lesion were manually definited by two masked retinal experts (authors EB and PV) in each CC en face image (Fig. 2B). Interobserver agreement (average) was found to be excellent in the MNV borders assessment (0.91 (confidence interval 0.88–0.92)). Consequently, the automated software outputted the MNV area (the entire area inside the drawn ROI) and the MNV flow area (the area of flow in the user-defined MNV lesion area) before and after loading phase (LP) of anti-VEGF in both groups (Fig. 3).

#### Central macular thickness (CMT)

CMT was calculated with the Optovue software. It was computed using the ETDRS grid system centered on the fovea in the central 1 mm-diameter circle (innermost ring/fovea).

### Statistical analysis

Statistical calculations were performed using (SPSS IBM Statistic 25, Chicago, IL). Distribution analysis of data was determined by Shapiro–Wilk test. Paired t-test was used for quantitative data analysis before and after LP injections in both groups. Friedman nonparametric test was employed to compare delta changes between groups. A p-value of less than 0.05 was chosen to be statistically significant.

## Data Availability

The datasets used and/or analyzed during the current study available from the corresponding author on reasonable request.
